# *In vitro* Anticoccidial Study of Oregano and Garlic Essential Oils and Effects on Growth Performance, Fecal Oocyst Output, and Intestinal Microbiota *in vivo*

**DOI:** 10.3389/fvets.2020.00420

**Published:** 2020-07-31

**Authors:** Erasmia Sidiropoulou, Ioannis Skoufos, Virginia Marugan-Hernandez, Ilias Giannenas, Eleftherios Bonos, Kelsilandia Aguiar-Martins, Diamanto Lazari, Damer P. Blake, Athina Tzora

**Affiliations:** ^1^Laboratory of Nutrition, School of Veterinary Medicine, Faculty of Health Sciences, Aristotle University of Thessaloniki, Thessaloniki, Greece; ^2^Laboratory of Animal Production, Nutrition and Biotechnology, Department of Agriculture, School of Agriculture, University of Ioannina, Arta, Greece; ^3^Department of Pathobiology and Population Sciences, Royal Veterinary College, University of London, Hertfordshire, United Kingdom; ^4^Laboratory of Pharmacognosy, School of Pharmacy, Faculty of Health Sciences, Aristotle University of Thessaloniki, Thessaloniki, Greece

**Keywords:** oregano essential oil, garlic essential oil, broiler chicken, performance, coccidia

## Abstract

This study investigated the *in vitro* effects of Greek oregano and garlic essential oils on inhibition of *Eimeria* parasites and their *in vivo* effects on production performance, intestinal bacteria counts, and oocyst output. An inhibition assay was performed *in vitro* using *Eimeria tenella* Wisconsin strain sporozoites and Madin-Darby bovine kidney (MDBK) cells. Intracellular sporozoite invasion was quantified by detection of *E. tenella* DNA using qPCR from cell monolayers harvested at 2 and 24 h post-infection. Parasite invasion was inhibited by the oregano essential oil at the concentration of 100 μg/ml by 83 or 93% after 2 or 24 h, respectively. Garlic essential oil reached a maximum inhibition of 70% after 24 h with the 50 μg/ml concentration. Normal morphology was observed in MDBK cells exposed to concentrations of 100 μl/ml of garlic or oregano for over 24 h. In the *in vivo* trial, 180 male broiler chicks (45.3 ± 0.7 g) were allocated into two treatments (6 pens of 15 chicks per treatment). Control treatment was fed commercial diets without antibiotics or anticoccidials. The ORE–GAR treatment was fed the same control diets, further supplemented with a premix (1 g/kg feed) containing the oregano (50 g/kg premix) and garlic (5 g/kg premix) essential oils. At day 37, all birds were slaughtered under commercial conditions, and intestinal samples were collected. ORE-GAR treatment had improved final body weight (1833.9 vs. 1.685.9 g; *p* < 0.01), improved feed conversion ratio (1.489 vs. 1.569; *p* < 0.01), and reduced fecal oocyst excretion (day 28: 3.672 vs. 3.989 log oocysts/g, *p* < 0.01; day 37: 3.475 vs. 4.007 log oocysts/g, *p* < 0.001). In the caecal digesta, ORE-GAR treatment had lower total anaerobe counts (8.216 vs. 8.824 CFU/g; p < 0.01), whereas in the jejunum digesta the ORE-GAR treatment had higher counts of *E. coli* (5.030 vs. 3.530 CFU/g; *p* = 0.01) and *Enterobacteriaceae* (5.341 vs. 3.829 CFU/g; *p* < 0.01), and lower counts of *Clostridium perfringens* (2.555 vs. 2.882 CFU/g; *p* < 0.01). In conclusion, the combined supplementation of oregano and garlic essential oils had a potent anticoccidial effect *in vitro* and a growth-promoting effect in broilers reared in the absence of anticoccidial drugs.

## Introduction

In the last few decades, the performance of broiler chickens has shown a continuous improvement in terms of achieving slaughter body weight in a shorter time, with improved feed efficiency. The main reasons for this achievement have been advances in modern broiler genotypes and intensification of management care and welfare. This remarkable improvement has been achieved in the European Union (EU) even after a ban on use of antibiotic growth promoters since 2006. However, the use of anticoccidial substances and ionophores is still permitted in broiler feeds within the EU. It must be noted that a different legislation frame exists in Canada, the USA, and some other parts of the world regarding drug-free broiler chicken production, where use of anticoccidial as well as antibacterial drugs is restricted (1, 2). This situation represents a great challenge to the poultry industry; coccidiosis persists as a problem and remains a major predisposing factor for the occurrence of necrotic enteritis and other pathogenic diseases (3, 4).

Among the most important areas of performance improvement for broiler chickens has been the advance in the use of feed additives with beneficial bioactive compounds that can protect against bacteria and parasites. One potential source of bioactive compounds that could be used as a natural growth enhancer with potent anticoccidial properties is oregano (*Origanum vulgare* subsp. *hirtum*), an aromatic-medicinal plant grown in Mediterranean countries (5, 6). Oregano and its main bioactive compounds have been shown to have positive antioxidant effects, a significant impact on intestinal microbiota and intestinal cell functionality (7–9). However, *in vitro* studies with oregano have been limited, primarily examining its antibacterial and antioxidant effects by conventional or spray drying microencapsulation techniques, without fully quantifying its anticoccidial properties (10–14). Nowadays, oregano may serve as a principal functional ingredient both in the food and feed industries, as well as in veterinary medicine (15, 16).

Another medicinal-aromatic plant with potential performance-enhancing, antioxidant, and anticoccidial activities is garlic (*Allium sativum*) (17–19). Garlic metabolites, when fed to broiler chicks, have been reported to increase resistance to experimental *Eimeria acervulina* infection and induce major alterations in broiler intestinal lymphocyte transcription in multiple networks including immune-related and cardiovascular-related gene pathways (20). It has been suggested that dietary immunomodulation by bioactive garlic ingredients may indicate a potential substitute to current drug-based strategies for commercial poultry production. The main compounds in garlic are organosulfurs, whose precursors (allicin, diallyl sulfide, and diallyl trisulfide) are believed to play key roles in antioxidant and anti-inflammatory pathways. These compounds appear to present potent anticoccidial activity and, coincidentally, an anti-inflammatory activity protecting host tissue from injuries induced by parasites (21). In some trials, dietary garlic supplementation limited or eliminated the negative effects of coccidial infection and improved average daily gain in challenged birds (19, 22). However, the beneficial results of garlic supplementation on production of healthy broilers has been variable, possibly as a result of heterogeneity in product composition, supplementation dose, nature of processing, and duration of feeding (23, 24). Moreover, the potential interactions between different combinations of bioactive ingredients have not been studied in depth.

The experimental work described here was designed to evaluate the combined dietary use of Greek oregano and garlic essential oils, both *in vitro* against chicken coccidial infection and *in vivo* in broiler chickens reared under farm conditions, in the absence of anticoccidial drugs.

## Materials and Methods

### Source of the Plant Essential Oils

The oregano leaves and flowers, and garlic roots and plants, were kindly donated by Mr. Fotis Stavratis, “Aromata Epirus,” Palaiohori, Filiates Thesprotia, Epirus, Greece.

The plant materials were submitted to hydrodistillation for 2 h using a modified Clevenger-type apparatus with a water-cooled oil receiver to reduce hydrodistillation overheating artifacts. The volatiles were trapped in 5 ml gas chromatography-grade *n*-hexane, according to the standard procedure described in European Pharmacopeia (25), dried over anhydrous sodium sulfate, and kept in closed, air-tight Pyrex containers at −4°C until use in the *in vitro* and *in vivo* trials.

The volatile constituents of the essential oils were analyzed by gas chromatography-mass spectrometry (GC-MS) analysis, using a Shimadzu GC-2010-GCMS-QP2010, as described previously in Giannenas et al. (26). Authentic compounds (Fluka, Sigma) were used for co-chromatography comparison.

### *In vitro* Trial

#### Essential Oils

Stocks of garlic and oregano essential oils were prepared to a final concentration of 1 mg/ml in DMSO (dimethyl sulfoxide).

#### Cell Culture

Madin-Darby bovine kidney (MDBK) cells (Sigma-Aldrich, UK) were maintained at 37°C−5% CO_2_ in Advanced DMEM (Gibco, Leicestershire, UK) supplemented with 2% fetal bovine serum (FBS; Sigma, Suffolk, UK) and 100 U penicillin/streptomycin (Fisher, Leicestershire, UK). Monolayers of MDBK cells were prepared in 24-well plates at 0.3 × 10^6^ cells/well and seeded ~3 h before infections.

#### Parasites

Sporozoites of the *E. tenella* Wisconsin strain (27) were used to perform infections. Oocyst excystation and sporozoite purification were performed as described previously in Pastor-Fernandez et al. (28).

#### Cytotoxicity Test

MDBK cells were incubated for over 24 h with a concentration of 100 μg/ml (the maximum to which cells were subsequently exposed during the invasion experiments) of oregano or garlic essential oils, modifying the approaches described in Pastor-Fernandez et al. (29). No morphological changes were observed compared with the control groups (DMEM/DMSO).

#### Pre-treatment

*Eimeria tenella* sporozoites (0.5 × 10^6^/well) were pre-treated for 1 h at 41°C−5% CO_2_ with essential oils of garlic or oregano at different concentrations (100, 50, 20, 5 μg/ml from the stocks) in Advanced DMEM, as described in Pastor-Fernandez et al. (29). DMSO (10 μl/ml) and robenidine (anticoccidial; 5 μg/ml) were used as controls for *E. tenella* infection.

#### Infection

After the pre-treatment, sporozoites were added to infect MDBK monolayers (41°C−5% CO_2_, 2 wells/time-point/condition), as described in Pastor-Fernandez et al. (29). At 2 and 24 h post-infection (HPI), infected monolayers were washed in phosphate-buffered saline (0.5 ml/well) and cells were dissociated with 0.3 ml Trypsin–EDTA. After centrifugation (10 min/1,000 × g), the pellet of each sample was retained and resuspended in 0.2 ml Proteinase K/PBS (1:10) and stored at −20°C.

#### Isolation of Nucleic Acids and Real-Time Quantitative PCR

Genomic DNA (gDNA) was isolated using a DNeasy Blood and Tissue Kit (Qiagen), according to company instructions. The DNA was eluted in a final volume of 165 μl per sample. Real-time quantitative PCR (qPCR) was performed in a CFX96 Touch Real-Time PCR Detection System (Bio-Rad, Hertfordshire, UK) according to Marugan-Hernandez et al. (30). Briefly, the quantification of *E. tenella* genomes per sample used gDNA and primers specific for the *Eimeria* genus 5S rDNA (Fw_5S: TCATCACCCAAAGGGATT and Rv_5S: TTCATACTGCGTCTAATGCAC) (31). Each qPCR plate used a mix of 19 μl/well (10 μl of Ssofast Eva Green, 0.5 μl of each 5S primer (10 μM), and 8.5 μl of water) and 1 μl of DNA. Serial dilutions of sporozoite gDNA equivalent to 1 × 10^7^ to 1 × 10^3^ genomes were included to produce a standard curve for genome quantification. All groups and the standard curve were evaluated testing three technical replicates per sample.

#### Data and Statistical Analysis

All data were analyzed using Bio-Rad CFX Manager software (Bio-Rad). The quantification of number of parasites was performed considering the standard deviation (SD) of Cq values, excluding SD >0.05. The average of values Starting Quantity (SQ) per sample was used to plot graphics seen in results. Statistical analysis was done by GraphPad (GraphPad Prism 8, California, USA). The Shapiro-Wilk test was used to access data normality. Differences and comparisons among groups were performed by one-way ANOVA or Kruskal-Wallis test, followed by Dunnett‘s multiple comparisons test.

Alternatively, the relative level of inhibition of *E. tenella* among groups treated with essential oil was assessed by a method adapted from Thabet et al. (32). The proportion of invasion or reproduction of parasites was calculated normalizing samples where DMSO was used to characterize the inhibition level:

$$\begin{array}{l} \text{Level of inhibition}\left(\text{\%}\right)=100\times \left(1-\frac{Average\;number\;of\;E.\;tenella\;genomes\;in\;treated\;sample}{Average\;number\;of\;E.\;tenella\;genomes\;in\;sample\;treated\;with\;DMSO}\right) \end{array}$$

### *In vivo* Trial

#### Animals, Diets, and Experimental Design

The trial protocol was authorized by the Research Project Innochicken and was co-financed by the European Regional Development Fund under the Operational Program “Epirus 2014–2020,” NSRF 2014–2020. Project Code: HΠ1AB-0028192.

Throughout the trial, the birds were handled in compliance with local laws, ethical practices, and regulations (33) and in accordance to the principles and guidelines for poultry welfare (34).

One hundred eighty 1-day-old male Ross-308 chicks (initial body weight 45.3 ± 0.7 g) were procured from PINDOS APSI hatchery and housed at a commercial poultry farm in Gavria, Arta (latitude 38.617°, longitude 20.767°), Epirus, Greece, during the period of October–December 2019. Each treatment group consisted of 6 replicate pens (length 1.0 m; width 1.1 m) of 15 chicks each. The stocking density was 15 birds/m^2^ (area of 1.1 m^2^ per pen). During the trial, commercial breeding and management procedures were employed; natural and artificial light was provided on a basis of 23 h for the first 2 days, 16 h from day 3 to day 14, and 21 h from day 15 to slaughter. Ambient temperature and humidity were controlled. All birds were vaccinated against Newcastle disease, infectious bronchitis, and infectious bursal disease (Gumboro) at the hatchery. Feed and drinking water were offered to all birds *ad libitum* throughout the experiment.

CONTROL group chickens were fed commercial typical corn and soybean meal-based rations in mash form which did not contain anticoccidials or antibiotics. The diets of the experimental ORE-GAR group were further supplemented at 1 g/kg feed, with a premix containing the essentials oils, throughout the trial. This premix was created using 50 g/kg oregano essential oil and 5 g/kg garlic essential oil, plus carrier (calcium carbonate).

Individual body weight was recorded on days 1, 12, 24, and 37. Feed consumption and mortality were recorded daily. At the end of the trial (day 37), all birds were slaughtered under commercial conditions. From each replicate pen, four birds were randomly selected and further processed. During necropsy of the selected birds, the gastrointestinal tract was removed for further analysis.

#### Gastrointestinal Tract Sampling

The abdomen of each chicken was cleaned with 70% (*v*/*v*) ethanol and skin incisions were made to give good access to the intestine. The caeca and jejunum of each bird were carefully removed and opened using a sterile scalpel, which then was used to gently scrape off the mucus layer from the intestinal content of each site and transfer it to a sterile container for further analysis.

#### Bacterial Cultivation and Bacterial Count

From each chicken, 1 g of intestinal content was homogenized with 9 ml of sterile peptone water solution 0.1%. For bacterial enumeration, Miles and Misra Plate Method (surface drop) was used, and each sample was diluted serially via 12-fold dilutions (from 10^−1^ to 10^−12^) using standard 96-well plates for microdilutions. Then 10 μl of each dilution was inoculated on media and incubated at 37°C for 48 h (35). MacConkey agar (Merck, Darmstadt, Germany) was used for the isolation and enumeration of *E. coli*. All plates were incubated aerobically at 37°C for 24–48 h. De Man, Rogosa, and Sharpe (MRS) agar (Oxoid, Basingstoke, UK) and TSC agar (Merck, Darmstadt, Germany) were used for the isolation and enumeration of *Lactobacilli* and *Clostridium perfringens*, while media were incubated at 30°C for 48 h and at 37°C for 24–48 h in anaerobic conditions, respectively. Total aerobic and anaerobic counts were determined using standard plate count agar medium (Oxoid) while plates were incubated at 30°C aerobically for 24–48 h and at 37°C anaerobically for 48–72 h, respectively. For the detection and enumeration of *Enterobacteriaceae*, Violet Red Bile Glycose (VRBG) agar was used and plates were incubated at 37°C aerobically for 24–48 h. For bacterial count, typical colonies from an appropriate dilution were counted and counts were expressed as CFU × log per 1 g wet weight sample. Typical colonies grown on media were then described and subcultured. Typical colonies from each medium were counted and colony-forming units per gram (CFU/g) of jejunum and cecal contents were calculated based on the given dilution. Typical colonies were selected for pure cultivation and confirmed into species level by VITEK 2 system (bioMérieux, Marcy l'Etoile, France) (36).

#### Coccidial Oocyst Count

At days 28 and 37, fresh fecal samples were collected from all experimental pens for assessment of coccidial oocyst output. The McMaster technique was used to detect and count coccidian oocysts in fecal samples (37, 38). *Eimeria acervulina* unsporulated oocysts were identified according to morphological features (oocyst shape, mean oocyst length, and mean oocyst width after microscopic examination).

#### Statistical Analysis

The basic study design was RCB (random complete block design) and the replication (pen) was considered as the experimental unit. Experimental data were analyzed by one-way ANOVA (general linear model) function of the SPSS statistical package (version 20.0) (39). Microbiology data were log-transformed (log10) before analysis. Data homogeneity was tested using Levene's test. Significance level was set at 5% (*p* ≤ 0.05). Values of *p* between 5 and 10% (0.05 < *p* ≤ 0.10) were reported as trends.

## Results

### Distillation of Essential Oils

The hydrodistillation of oregano fresh material yielded 5.49% essential oil. Respectively, the hydrodistillation of the garlic plant material yielded 0.15% essential oil.

The major compounds identified in the oregano essential oil (Table 1) were carvacrol (57.95%), γ-terpinene (10.59%), and *p*-cymene (8.90%). Moreover, the major compounds identified in the garlic essential oil (Table 2) were diallyl trisulfide (58.46%) and diallyl disulfide (24.54%).

**Table 1:** Oregano essential oil composition analyzed by gas chromatography–mass spectrometry.

	**Compounds^a^**	**RT**	**%**	**Identification^b^**
1	α-Pinene	4.399	0.52	RT, MS, Co-GC
2	α-Thujene	4.534	0.83	RT, MS, Co-GC
3	Camphene	5.510	0.07	RT, MS
4	α-Phellandrene	9.863	0.23	RT, MS
5	β-Myrcene	10.210	2.03	RT, MS, Co-GC
6	α-Terpinene	10.808	1.84	RT, MS, Co-GC
7	γ-Terpinene	16.641	10.59	RT, MS, Co-GC
8	p-Cymene	19.608	8.90	RT, MS, Co-GC
9	1-Octen-3-ol	34.963	0.61	RT, MS
10	*cis*-Sabinenehydrate	35.365	0.34	RT, MS
11	β-Caryophyllene	40.800	1.01	RT, MS, Co-GC
12	1-Terpinen-4-ol	41.688	0.37	RT, MS, Co-GC
13	Thymol methyl ether	41.829	0.21	RT, MS
14	Borneol	45.772	0.40	RT, MS, Co-GC
15	β-Bisabolene	46.715	0.41	RT, MS, Co-GC
16	Thymol	62.680	3.69	RT, MS, Co-GC
17	Carvacrol	63.512	67.95	RT, MS, Co-GC

a *Compounds are listed in order of elution from an INNOWAX capillary column*.

b *RT, retention time; MS, mass spectrum; Co-GC, coinjection with authentic compound*.

**Table 2:** Garlic essential oil composition analyzed by gas chromatography-mass spectrometry.

	**Compounds^a^**	**RT**	**%**	**Identification^b^**
1	D-Limonene	11.894	0.09	RT, MS, Co
2	Eucalyptol	12.496	0.05	RT, MS, Co
3	*p*-Cymene	19.112	0.14	RT, MS, Co
4	Dimethyl trisulfide	34.473	1.25	RT, MS
5	Diallyl disulfide	35.465	24.54	RT, MS
6	Diallyl tetrasulfide	35.718	4.73	RT, MS
7	Camphor	36.590	0.05	RT, MS, Co-GC
8	*N, N*-Dimethyl-ethanethioamide	37.233	0.63	RT, MS
9	Linalool	39.598	0.07	RT, MS, Co-GC
10	Linalyl butyrate	39.738	0.04	RT, MS
11	Allyl methyl trisulfide	40.443	4.42	RT, MS
12	3-Vinyl-1,2-dithiocyclohex-4-ene	46.417	0.17	RT, MS
13	Diallyl trisulfide	48.746	58.46	RT, MS
14	3-Vinyl-1,2-dithiocyclohex-5-ene	50.561	0.64	RT, MS
15	(methylsulfinyl)(methylthio)-Methane	51.461	0.24	RT, MS
16	3-(Methylthio)pent-4-yn-1-ol	52.233	0.11	RT, MS
17	*cis*-2-Thiabicyclo[3.3.0]octane	54.617	0.06	RT, MS
18	Isobutyl isothiocyanate	58.125	0.08	RT, MS
19	Epiglobulol	58.962	0.18	RT, MS
20	Butyl isothiocyanate	59.251	0.02	RT, MS
21	*p*-Cymen-7-ol	62.505	0.05	RT, MS
22	Hinesol	62.896	0.16	RT, MS
23	Carvacrol	63.291	1.22	RT, MS
24	Patchoulane	66.673	0.15	RT, MS
25	Apiol	67.266	0.26	RT, MS
26	1-Docosanol	72.058	0.12	RT, MS

a *Compounds are listed in order of elution from an INNOWAX capillary column*.

b *RT, retention time; MS, mass spectrum; Co-GC, coinjection with authentic compound*.

### *In vitro* Trial

**Figure 1:**
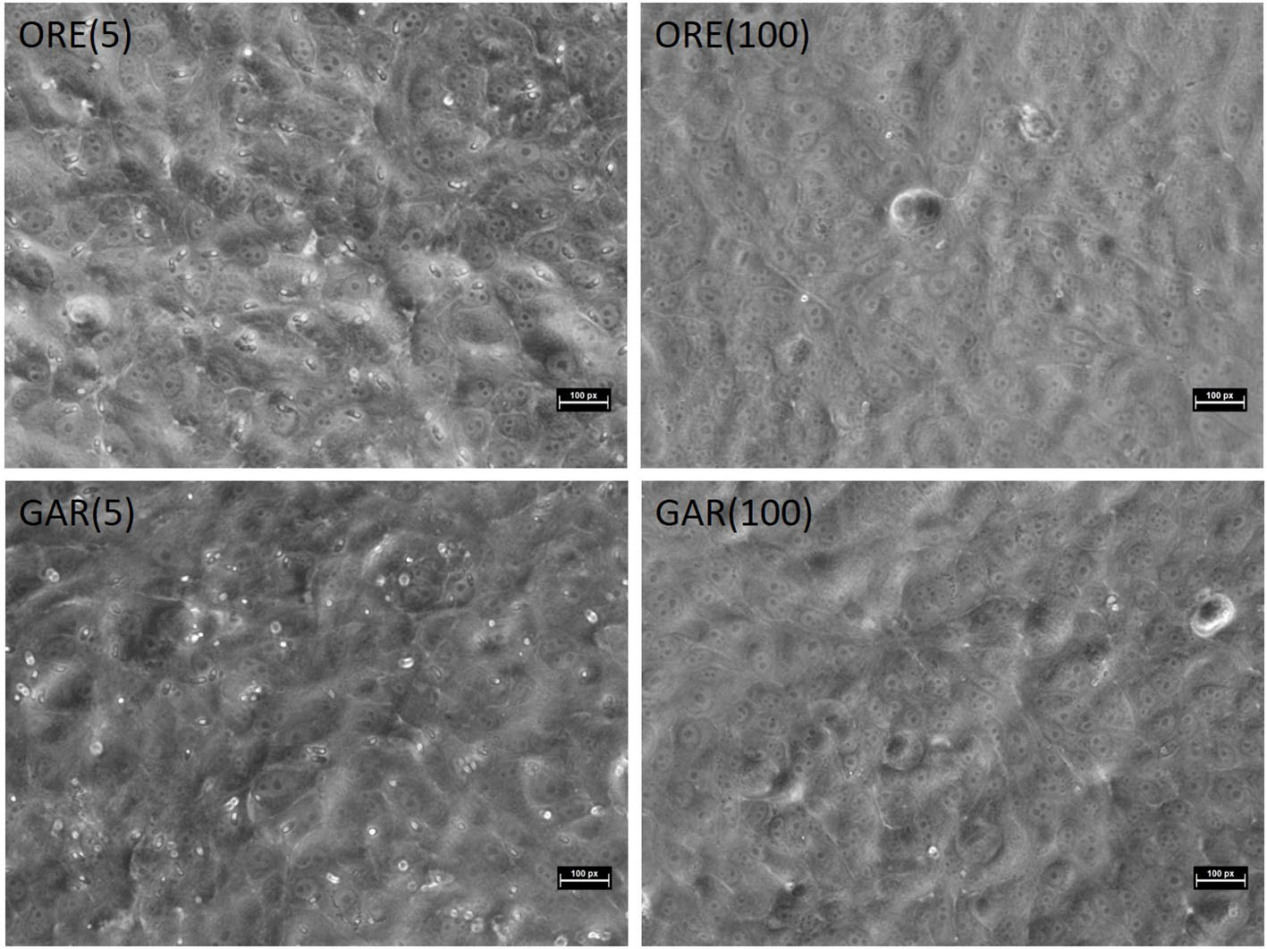
MDBK monolayers infected with pre-treated sporozoites at 24 HPI. Pre-treatment with the lowest doses of essential oils showed little reduction in infection; almost no intracellular sporozoites were found when the highest doses were used. MDBK morphology was not affected by the treatment. ORE, oregano; GAR, garlic; (5), 5 μg/ml; (100), 100 μg/ml. Bars ~30 μm.

**Figure 2:**
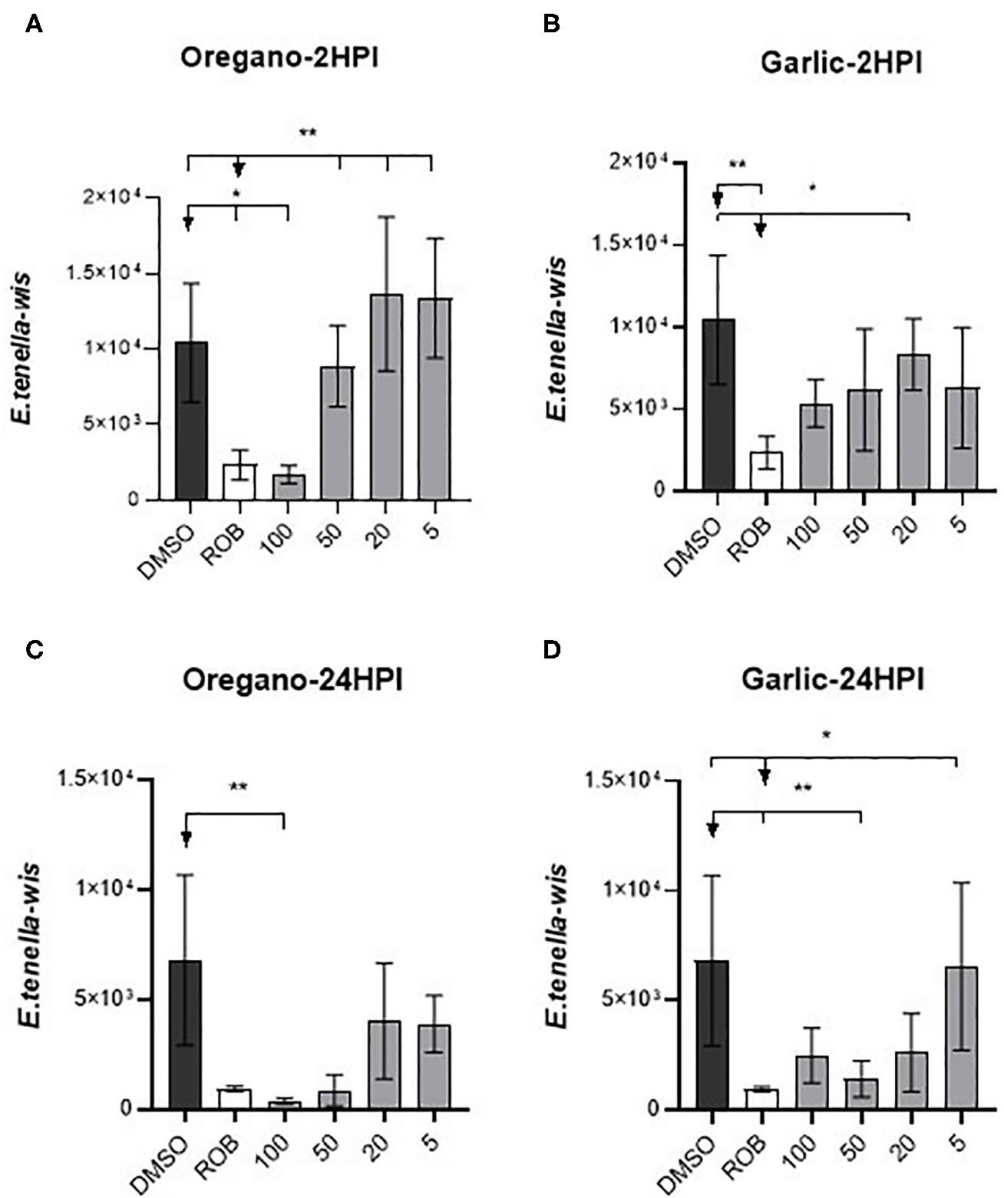
Effect of essential oils on *E. tenella* infection of MDBK. Intracellular number of parasites: **(A)** 2 HPI after pre-treatment with oregano essential oil; **(B)** 2 HPI after treatment with garlic essential oil; **(C)** 24 HPI after treatment with oregano essential oil; **(D)** 24 HPI after treatment with garlic essential oil. X-axis numbers represent the dilution in μg/ml used. DMSO, dimethyl sulfoxide (10 μl/ml); ROB, robenidine (5 μg/ml). Asterisks indicate the significance among groups (Dunnett's multiple comparisons test, *p* < 0.05), whereas *groups compared with ROB and **groups compared with DMSO.

As shown in Figure 1, parasite invasion and replication was not notably influenced by pre-treatment with the lowest doses of either essential oil, but almost no intracellular sporozoites were found when the highest doses were used. MDBK morphology was not affected by either treatment, even at higher levels of exposure. *In vitro* evaluation of sporozoite invasion in MDBK cells after treatments showed differences among groups testing both oils at 2 HPI (one-way ANOVA test, *p* < 0.05). Oregano essential oil at 100 μg/ml significantly inhibited sporozoite invasion (as much as robenidine control inhibition) when compared with DMSO (Dunnett's multiple comparisons test, *p* < 0.05) (Figure 2A). Lower concentrations of oregano essential oil (50, 10, and 5 μg/ml) also exhibited an effect on sporozoite invasion; however, the difference was not significant when compared with the DMSO control (Dunnett's multiple comparisons test, *p* < 0.05). In a similar way, inhibition of invasion at 24 HPI using oregano was only significant for sporozoites pre-treated with 100 μg/ml (Dunnett‘s multiple comparisons test, *p* < 0.05) (Figure 2C). The effect of garlic essential oil at 2 HPI was less intense than oregano (Figure 2B), but significant inhibition was still observed compared with DMSO control when 20 μg/ml was used during sporozoite pre-incubation (Dunnett's multiple comparisons test, *p* < 0.05). Nonetheless, by 24 HPI, variation in sporozoite inhibition became statistically significant when 50 or 100 μg/ml was used for pre-incubation (Figure 2D).

The normalization of intracellular parasite numbers in each group with DMSO control was also assessed to dismiss a potential effect caused by the solvent. Proportions of relative inhibition of sporozoites are shown in Table 3 and agree with the previous analysis. Pre-treatment with oregano essential oil at 100 μg/ml after 24 HPI presented the highest inhibition among all treatments, reducing the number of intracellular parasites by ~93%. The maximum inhibition following pre-treatment with garlic essential oil was 70% after 24 HPI when using 50 μg/ml.

**Table 3:** Effect of oregano and garlic essential oils on relative inhibition of *E. tenella* in MDBK cells at 2 and 24 HPI.

**Time point/treatment (μg/ml)**	**ROB**	**GAR (100)**	**GAR (50)**	**GAR (20)**	**GAR (5)**	**ORE (100)**	**ORE (50)**	**ORE (20)**	**ORE (5)**
2 h	76.07 ± 11.80	46.54 ± 15.30	44.70 ± 29.82	17.04 ± 24.12	41.21 ± 10.99	82.89 ± 6.95	18.54 ± 37.09	0	0
24 h	83.39 ± 13.14	55.94 ± 24.81	70.37 ± 41.34	62.63 ± 4.12	12.99 ± 25.98	92.99 ± 6.90	81.57 ± 25.60	38.14 ± 13.17	33.38 ± 66.76

*ROB, robenidine; GAR, garlic essential oil; ORE, oregano essential oil*.

*(100) (50) (20) (5): concentration in μg/ml*.

*Average (two biological replicates) ± range*.

### *In vivo* Trial

The effects of essential oil supplementation on broiler chicken performance are shown in Table 4. Live body weight was increased (*p* < 0.01) in the supplemented ORE-GAR treatment on day 12 of the trial, and this increase was significant (*p* < 0.01) on all other comparisons. The final body weight (day 71) was increased (*p* < 0.01) by 148.0 g in the ORE-GAR treatment. Feed intake did not differ (*p* > 0.10) for the examined periods and overall. Feed conversion ratio was significantly improved (*p* < 0.01) during the period from day 13 to 24, as well as for the overall trial (*p* < 0.01).

**Table 4:** Effect of oregano and garlic dietary supplementation on *in vivo* broiler chicken performance parameters.

**Live body weight**	**CONTROL**	**ORE-GAR**	**SEM**	***P*-value**
**on day (g)**	**treatment**	**treatment**		
1	45.0	45.2	0.14	0.598
12	303.6^x^	314.4^y^	2.85	0.087
24	923.1^a^	1016.6^b^	14.56	0.009
37	1685.9^a^	1833.9^b^	18.78	0.003
**Feed intake during period (g)**				
1–12 days	322.7	323.8	0.00	1.000
13–24 days	1007.9	1004.1	5.93	0.752
25–37 days	1224.6	1318.9	28.63	0.131
1–37 days	2574.8	2662.6	34.34	0.230
**Feed conversion ratio during period (g feed/g weight gain)**				
1–12 days	1.249	1.204	0.014	0.132
13–24 days	1.630^b^	1.439^a^	0.029	0.009
25–37 days	1.608	1.614	0.035	0.927
1–37 days	1.569^b^	1.489^a^	0.012	0.009

*Number of replicates = each treatment had 6 pens of 15 male birds/pen*.

*SEM, standard error of mean*.

a,b *Values in the same row without superscripts in common differ significantly (p ≤ 0.05)*.

x,y *Values in the same row without superscripts in common tend to differ (0.05 < p ≤ 0.10)*.

In Table 5, the effects of essential oil supplementation on the examined intestinal microflora populations are presented. In the caeca, total anaerobes were found to be lower (*p* < 0.01) in the ORE-GAR treatment compared with the CONTROL, but no differences (*p* > 0.10) were noted for the other examined populations (total aerobes, *E. coli, Enterobacteriaceae, Lactobacillus, C. perfringens*). In the jejunum, the ORE-GAR treatment group presented higher counts of *E. coli* (*p* = 0.01), *Enterobacteriaceae* (*p* < 0.01), and lower counts of *C. perfringens* (*p* < 0.01) compared with the CONTROL treatment, but no differences were noted for the other examined populations (total aerobes, total anaerobes, *Lactobacillus* spp.).

**Table 5:** Effect of oregano and garlic dietary supplementation on *in vivo* broiler chicken intestinal populations.

	**CONTROL**	**ORE–GAR treatment**	**SEM**	***P*-value**
	**treatment**	**treatment**		
**Cecum microbial populations (log**_**10**_ **CFU/g digesta)**				
Aerobes (PCA)	7.751	8.239	0.170	0.182
Anaerobes (PCA)	8.824^b^	8.216^a^	0.082	0.004
*E. coli* (McC)	7.440	7.172	0.136	0.349
*Enterobacteriaceae* (VRBG)	7.564	7.289	0.116	0.261
*Lactobacillus* (MRS)	8.251	8.271	0.212	0.964
*Clostridium perfringens* (TSC)	4.869	4.816	0.209	0.902
**Jejenum microbial populations (log**_**10**_ **CFU/g digesta)**				
Aerobes (PCA)	6.263	6.405	0.205	0.736
Anaerobes (PCA)	8.143	7.851	0.147	0.346
*E. coli* (McC)	3.530^a^	5.030^b^	0.235	0.010
*Enterobacteriaceae* (VRBG)	3.829^a^	5.341^b^	0.192	0.003
*Lactobacillus* (MRS)	7.576	7.562	0.205	0.975
*Clostridium perfringens* (TSC)	2.882^b^	2.555^a^	0.038	0.002

*Number of replicates = each treatment had 6 pens of 15 male birds/pen*.

*SEM, standard error of mean*.

a,b *Values in the same row without superscripts in common differ significantly (p ≤ 0.05)*.

As seen in Table 6, fecal oocyst counts were affected by the dietary essential oil supplementation. On day 28, the ORE-GAR treatment had significantly lower (*p* < 0.01) counts by 0.317 units (log10/g feces), and on day 37 it had significantly lower (*p* < 0.001) counts by 0.532 units (log10/g feces).

**Table 6:** Effect of oregano and garlic dietary supplementation on fecal oocyst output from broiler chickens reared under commercial conditions.

**Oocyst counts in feces**	**CONTROL**	**ORE–GAR**	**SEM**	***P*-value**
**(log_**10**_ CFU/g feces)**	**treatment**	**treatment**		
Day 28	3.989^b^	3.672^a^	0.046	0.006
Day 37	4.007^b^	3.475^a^	0.048	<0.001

*Number of replicates = each treatment had 6 pens of 15 male birds/pen*.

*SEM, standard error of mean*.

a,b *Values in the same row without superscripts in common differ significantly (p ≤ 0.05)*.

## Discussion

Coccidiosis remains a severe challenge for the broiler industry owing to the ubiquity of *Eimeria* species parasites and the widespread occurrence of anticoccidial resistance. Today, resistance to anticoccidial drugs and a broad spectrum of other products is easily acquired. Further, consumer opinion is now commonly demanding reduced use of drugs in livestock production, including ionophores or chemical coccidiostats, as well as antibiotic growth promoters (40). As restrictions on the use of anticoccidial drugs increase, and in the absence of affordable or scalable anticoccidial vaccines for broilers, research exploring safe and effective alternatives to control coccidiosis has increased leading to the exploitation of herbal extracts such as essential oils of aromatic or medicinal plants (16, 41). One example has been phenolic compounds, a large group of bioactive constituents found in a wide variety of plants that include thousands of compounds with different chemical structures (42, 43). Because of interest in their medicinal and sensorial properties (color and astringency), their analysis in foods, beverages, and feed has been developing in recent years. Similarly, multiple sources of compounds have been investigated because total phenolic content (expressed in mg/g) can be highly variable, for example, between 0.24 mg/g can be detected in grape seed extracts compared with 147 mg/g in basil extracts (44, 45). In our work, oregano and garlic essential oils were examined. Based on the GC-MS results, the main bioactive compounds of oregano essential oil were carvacrol, γ-terpinene, and *p*-cymene, whereas the major compounds of garlic essential oil were diallyl sulfides (diallyl trisulfide and diallyl disulfide). We evaluated the *in vitro* anticoccidial activity of both oregano and garlic essential oils with the inhibition of coccidial (*E. tenella*) invasion in MDBK cells. Moreover, in our *in vivo* trial, we examined the impact of diet supplementation with oregano and garlic essential oils on broiler growth performance, oocyst excretion after natural environmental challenge, and components of the intestinal microbiota.

The results showed that both oregano and garlic possess very strong anticoccidial activity *in vitro*, evidenced by the inhibition of sporozoite invasion at the higher concentrations tested, potentially caused by a toxic effect that left few parasites fit to invade cells. Oregano essential oil exhibited an effect comparable with robenidine, a well-known anticoccidiostat. The same high essential oil concentration did not show any deleterious effects on the host cells based upon a microscopic assessment of cell morphology within the monolayer. Cytotoxic effects on the host cells could have affected parasite invasion and proliferation (46). Sporozoites were shown to begin endogenous development into schizonts from 28 HPI. Further studies to evaluate the effects of both essential oils in this part of the eimerian lifecycle, and the extent to which pre-treatment of free sporozoites has an effect, would be of great interest. Although the mode(s) of action or mechanisms involved have not been elucidated, a reasonable explanation for this anticoccidial activity is the hydrophobic character and low molecular weight of the main phenolic compounds present in those essential oils that allow them to disintegrate outer cell membranes (47). This may cause an increase in cytoplasmic membrane permeability and lead to cell death caused by leakage of ions, energy loss, and diffusion of cell contents (11). Further, the high lipid solubility of oregano and garlic essential oils is likely to permit rapid diffusion through parasite and host cell membranes. Other possible mechanisms include interference with the calcium-mediated signaling that is a necessary mechanism for invasion by *E. tenella* sporozoites (47). The hydrophobic character of those compounds may suggest interaction with membrane components and permeability (48). If the carvacrol concentration increases, then more molecules interact with the phospholipid bilayer, upsetting membrane fluidity (49). Accordingly, carvacrol, thymol, and allicin, the major bioactive ingredients of the mixture tested here, may also exert a toxic effect on the upper layer of mature enterocytes of the intestinal mucosa. For this reason, we also assessed their cytotoxic effect *in vitro* and found it to be very low. Moreover, other studies have demonstrated that MDBK cells are able to produce cytokines when stimulated by exposure to viruses, suggesting that an immune response could also be involved in the anticoccidial effect of these metabolite compounds of oregano and garlic (10, 50).

Our *in vitro* results were complemented by *in vivo* findings with broiler chickens raised in the absence of in-feed anticoccidials or antibiotics. In our study, the baseline performance level of the broiler flock can be regarded as high, with apparently low environmental stress and limited pathogen challenge. Nonetheless, the group of chickens that received oregano and garlic essential oils demonstrated higher performance as indicated by weight gain, feed-to-gain ratio, and also by lower levels of fecal oocyst excretion. These results are consistent with previous studies with challenged broilers (5, 15, 22, 42).

In the last decade, the published literature has shown increased research interest in the use of aromatic plants' bioactive substances in the diets of meat-type animals as an alternative to decrease use of antimicrobial or anticoccidial drugs. Antibiotics have been widely used in veterinary medicine worldwide to treat bacterial animal diseases and to protect the health of farm animals. Important parameters of the use of antibiotics in veterinary medicine include their use, sales, exposure pathways, environmental occurrence, fate, and effects (4, 43). Many antimicrobials are poorly absorbed from the chicken intestine, leaving a large percentage to be excreted unchanged in feces. Animal waste is commonly used as a fertilizer in many countries, potentially leading to the spread of antimicrobials over large land areas and contributing to a growing global alarm about the adverse effects of antibiotic residues on the environment (44). According to WHO, there are now serious concerns about increasing antibiotic resistance gene carriage in microorganisms found in human patients, possibly as a result of the veterinary use of antimicrobials (45). In the current study, the broiler diets did not contain any antimicrobial or anticoccidial substances. Known for their therapeutic properties, medicinal plants may serve as good alternatives to recognized drugs (51). It has been reported that up to 80% of citizens from developed countries still use “traditional” medicine, for example, medicinal plants or their derived substances. Aromatic plants are still under investigation to better comprehend their medicinal properties, benefits, and safety (52). Plant extracts from plants such as oregano and garlic contain important bioactive phytochemicals, and many such substances are being shown to possess antimicrobial properties that could be used in therapeutic treatments. Thus, every year many studies are performed all over the globe to prove the efficiency of phytochemicals (47, 52, 53).

A major target in this study was to test whether the combination of oregano and garlic essential oils possess anticoccidial properties when used as feed ingredients, without the requirement for a withdrawal period and without the detrimental effects that can be associated with anticoccidial drugs. One of the most significant findings of the present study was that supplementation of broilers reared in commercial conditions with a mixture of oils of oregano and garlic reduced oocyst excretion compared with the control treatment at both examined time points (days 28 and 37). It is well-known that the bacterial cell wall is a primary target for the antibacterial effects of phenols. Building on our *in vitro* study, it is likely that phenolic compounds also exert their activity on intestinal pathogenic bacteria and protozoa *in vivo*, and on the intestinal cells of the host (22, 49, 54). It is worth noting that the effectiveness of incorporating plant-based extracts into broiler diets may be variable, and that further complications may arise because of the rich composition of the mixtures of the herbal feed additive, requiring further research.

Another implication of *Eimeria* infection, besides the direct consequences on animal health and welfare, is its impact on the enteric microbiota. Recently, it has been shown that the severity of pathology caused by *E. tenella* infection of broiler chickens as quantified by intestinal lesion scoring can be linked with changes in enteric microbial occurrence and population structure (55). In our trial, the diversity of microbial composition within caecal and jejunal microbiomes showed a potentially more beneficial composition in broilers fed the mixture of oregano and garlic oil compared with control chickens. The current study evaluated the composition and structure of specific caecal and jejunal bacteria in the absence of a defined *Eimeria* challenge. A significant increase in *Enterobacteriaceae* and *E. coli* was found in the jejunum of the essential oil-supplemented birds, but this effect was not repeated in the caeca. *Clostridium perfringens* is a ubiquitous organism, widely distributed in nature and in human and animal intestinal tracts where it presents in high numbers when specific predisposing factors influence digestive system integrity (56). Here, oregano and garlic essential oil supplementation lowered *C. perfringens* counts in the jejunum, a site where it has been associated with intestinal epithelium inflammation and stimulation of bacterial dysbiosis. However, no statistically significant variation was noted for *C. perfringens* occurrence in the caeca. In the literature, it has also been well-described that coccidiosis and stress are predisposing factors for alterations in the intestinal microflora and in *C. perfringens* occurrence (57, 58).

*Eimeria* infections have been found to increase the abundance of the genera *Escherichia, Shigella*, and *Klebsiella*, members of the *Enterobacteriaceae* family that have been described as opportunistic pathogens (55). In contrast, it has been reported that parasitic infection decreased the levels of *Lactobacillus*, many of which are regarded as beneficial for gut health (59, 60). These results of our study are in agreement with our previous studies, where similar herbal mixtures modified bacterial populations (8, 53, 61), despite the fact that some *Enterobacteriaceae* species of intestinal bacteria were not influenced substantially by the oregano and garlic feed additives.

**Figure 3:**
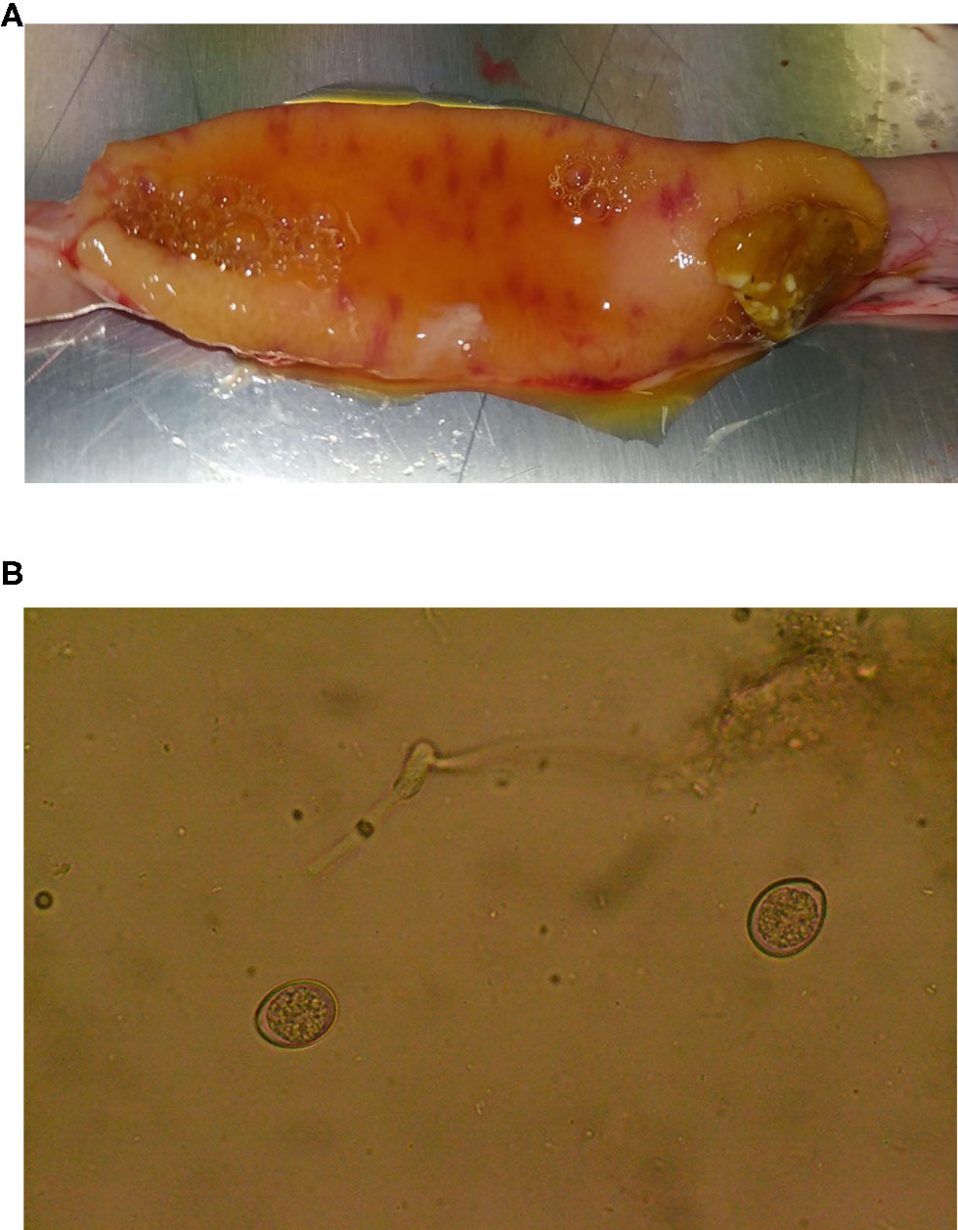
**(A)** Lesions in the small intestine of a 37-day-old chicken, associated with *Eimeria* parasites. **(B)** *Eimeria acervulina* oocysts found during microscopic examination of jejunal contents from a 37-day-old chicken.

The broiler chickens tested here were not exposed to experimental *Eimeria* infection. Instead, they were reared in a commercial setting where natural exposure was likely. During screening, *Eimeria* infection was detected, diagnosed at *E. acervulina* based on gross intestinal pathology (Figure 3A) and oocyst morphology (Figure 3B). Consideration of oocyst excretion indicated that parasite invasion and/or replication was reduced by the essential oil supplementation given that all other factors were identical (housing, stocking density, season, management).

## Conclusion

In conclusion, the results of the present study suggest that diet inclusion of oregano and garlic essential oils can improve growth performance in broiler chickens and reduce *Eimeria* oocyst output by exerting a coccidiostatic effect, supported by *in vitro* tests using *E. tenella*. Oregano and garlic essential oils exerted positive effects on the intestinal microbiota, supporting interactions between diet-mediated alterations in the microbiota and chicken growth and performance. Although it is difficult to extrapolate *in vitro* results to *in vivo*, the present study shows that *in vitro* inhibition of parasite invasion correlates well with *in vivo* findings. This study provides credible evidence that the hypothesis of rearing broilers without anticoccidial drugs or ionophores is possible. More extensive large scale studies and *in vivo* challenge trials are required to confirm this possibility.

## Data Availability Statement

The datasets presented in this article are not readily available because data will be available after the end of the project. Requests to access the datasets should be directed to igiannenas@vet.auth.gr.

## Ethics Statement

The animal study was reviewed and approved by Research Committee, Aristotle University of Thessaloniki, Greece.

## Author Contributions

All authors listed have made a substantial, direct and intellectual contribution to the work, and approved it for publication.

## Conflict of Interest

The authors declare that the research was conducted in the absence of any commercial or financial relationships that could be construed as a potential conflict of interest.

## References

[1] Gaucher M-L Quessy S Letellier A Arsenault J Boulianne M. Impact of a drug-free program on broiler chicken growth performances, gut health, *Clostridium perfringens* and *Campylobacter jejuni* occurrences at the farm level. Poult Sci. (2015) 94:1791–801. 10.3382/ps/pev142 26047674

[2] Giannenas I Bonos E Skoufos I Tzora A Stylianaki I Lazari D. Effect of herbal feed additives on performance parameters, intestinal microbiota, intestinal morphology and meat lipid oxidation of broiler chicken. Br Poult Sci. (2018) 59:545–53. 10.1080/00071668.2018.1483577 29873243

[3] Timbermont L Haesebrouck F Ducatelle R van Immerseel F. Necrotic enteritis in broilers: an updated review on the pathogenesis. Avian Pathol. (2011) 40:341–7. 10.1080/03079457.2011.590967 21812711

[4] Huyghebaert G Ducatelle R van Immerseel. F. An update on alternatives to antimicrobial growth promoters for broilers. Vet J. (2011) 187:182–8. 10.1016/j.tvjl.2010.03.003 20382054

[5] Tsinas A Giannenas I Voidarou C Tzora A Skoufos J Effects of an oregano based dietary supplement on performance of broiler chickens experimentally infected with *Eimeria acervulina* and *Eimeria maxima*. J Poultry Sci. (2011) 48:194–200. 10.2141/jpsa.010123

[6] Bozkurt M Giannenas I Kucukyilmaz K Christaki E Florou-Paneri P. An update on approaches to controlling coccidia in poultry using botanical extracts. Br Poult Sci. (2013) 54:713–27. 10.1080/00071668.2013.849795 24397508

[7] Giannenas I Bonos E Christaki E Florou-Paneri P Oregano: a feed additive with functional properties. In Holban AM. Grumezescu AM, editors. Therapeutic Foods, Handbook of Food Engineering, (Vol 8), London, UK: Elsevier Academic Press (2018). p. 179–208. 10.1016/B978-0-12-811517-6.00006-4

[8] Tzora A Giannenas I Karamoutsios A Papaioannou N Papanastasiou D Bonos E Effects of oregano, attapulgite, benzoic acid and their blend on chicken performance, intestinal microbiology and Intestinal morphology. J. Poultry Sci. (2017) 54:218–27. 10.2141/jpsa.0160071 PMC7477216 32908429

[9] Paraskeuas V Fegeros K Palamidi I Hunger C Mountzouris KC. Growth performance nutrient digestibility, antioxidant capacity, blood biochemical biomarkers and cytokines expression in broiler chickens fed different phytogenic levels. Anim Nutr. (2017) 3:114–20. 10.1016/j.aninu.2017.01.005 29767099 PMC5941105

[10] Burt SA Tersteeg-Zijderveld MHG Jongerius-Gortemaker BGM Vervelde L Vernooij JC. *In vitro* inhibition of *Eimeria tenella* invasion of epithelial cells by phytochemicals. Vet Parasitol. (2013) 191:374–8. 10.1016/j.vetpar.2012.09.001 23021265

[11] Ultee A Kets EPW Smid EJ. Mechanisms of action of carvacrol on the food-borne pathogen. Appl Environ Microb. (1999) 65:4606–10. 10.1128/AEM.65.10.4606-4610.1999 10508096 PMC91614

[12] Partheniadis I Vergkizi S Lazari D Reppas C Nikolakakis I Formulation, characterization and antimicrobial activity of tablets of essential oil prepared by compression of spray-dried powder. J Drug Deliv Sci Technol. (2019) 50:226–36. 10.1016/j.jddst.2019.01.031

[13] Da Costa SB Duarte C Bourbon AI Pinheiro AC Serra AT Martins MM Effect of the matrix system in the delivery and *in vitro* bioactivity of microencapsulated Oregano essential oil. J Food Eng. (2012) 110:190–9. 10.1016/j.jfoodeng.2011.05.043

[14] Lagouri V Blekas G Tsimidou M Kokkini S Boskou D Composition and antioxidant activity of essential oils from Oregano plants grown wild in Greece. Z Lebensm Unters Forch. (1993) 197:20–3. 10.1007/BF01202694

[15] Bozkurt M Ege G Aysul N Aksit H Tuzun AE Kucukyilmaz K. Effect of anticoccidial monensin with oregano essential oil on broilers experimentally challenged with mixed Eimeria spp. Poult Sci. (2016) 95:1858–68. 10.3382/ps/pew077 26976910

[16] Giannenas I Sidiropoulou E Bonos E Christaki E Florou-Paneri P The history of herbs, medicinal and aromatic plants, and their extracts: past, current situation and future perspectives. In Florou-Paneri P Christaki E Giannenas I, editors. Feed Additives: Aromatic Plants and Herbs in Animal Nutrition And Health. London, UK: Elsevier (2019) 1–8. 10.1016/B978-0-12-814700-9.00001-7

[17] Stanacev V Glamocic D Milosevic N Puvaca N Stanacev V Plavsa N Effect of garlic (*Allium sativum* L.) in fattening chicks nutrition. Afr J Agric Res. (2011) 6:943–8. 10.5897/AJAR10.908

[18] Sheoran N Kumar R Kumar AA Batra K Sihag S Maan S. Nutrigenomic evaluation of garlic (*Allium sativum*) and holy basil (*Ocimum sanctum*) leaf powder supplementation on growth performance and immune characteristics in broilers. Vet World. (2017) 10:121–9. 10.14202/vetworld.2017.121-129 28246456 PMC5301171

[19] Pourali M Kermanshahi H Golian A Ramzi GR Soukhtanloo M Antioxidant and anticoccidial effects of garlic powder and sulfur amino acids on *Eimeria*-infected and uninfected broiler chickens. Iran J Vet Res. (2014) 15:227–32. 10.22099/ijvr.2014.2531

[20] Kim DH Lillehoj H Lee S Lillehoj E Bravo D. Improved resistance to *Eimeria acervulina* infection in chickens due to dietary supplementation with garlic metabolites. Br J Nutr. (2013) 109:76–88. 10.1017/S0007114512000530 22717023

[21] Dkhil MA Abdel-Baki AS Wunderlich F Sies H Al-Quraishy S. Anticoccidial and antiinflammatory activity of garlic in murine *Eimeria papillata* infections. Vet Parasitol. (2011) 175:66–72. 10.1016/j.vetpar.2010.09.009 20943319

[22] Ali M Chand N Khan RU Naz S Gul S Anticoccidial effect of garlic (*Allium sativum*) and ginger (*Zingiber officinale*) against experimentally induced coccidiosis in broiler chickens. J. Appl Anim Res. (2019) 47:79–84. 10.1080/09712119.2019.1573731

[23] Khan RU Nikousefat Z Tufarelli V Naz S Javdani M Laudadio V Garlic (*Allium sativum*) supplementation in poultry diets: effect on production and physiology. Worlds Poult Sci J. (2012) 68:417–24. 10.1017/S0043933912000530

[24] Puvaca N Ljubojevic D Kostadinovic LJ Levic J Nikolova N Miscevic B Spices and herbs in broiler nutrition: hot red pepper (*Capsicum annuum* L.) and its mode of action. Worlds Poult. Sci. J. (2015) 71:683–8. 10.1017/S004393391500241X

[25] Council of Europe European Pharmacopoeia. 5th ed Strasbourg: COE (2005). p. 2710–1.

[26] Giannenas I Bonos E Filliousis G Stylianaki I Kumar P Lazari D Effect of a polyherbal or an arsenic-containing feed additive on growth performance of broiler chickens, intestinal microbiota, intestinal morphology and lipid oxidation of breast and thigh meat. J Appl Poultry Res. (2019) 28:164–75. 10.3382/japr/pfy059

[27] McDougald LR Jeffers TK. Comparative *in vitro* development of precocious and normal strains of *Eimeria tenella* (Coccidia). J Protozool. (1976) 23:530–4. 10.1111/j.1550-7408.1976.tb03834.x 1003341

[28] Pastor-Fernandez I Kim SJ Billington K Bumstead J Marugan-Hernandez V Kuster T. Development of cross-protective *Eimeria*-vectored vaccines based on apical membrane antigens. Int J Parasitol. (2018) 48:505–18. 10.1016/j.ijpara.2018.01.003 29524526

[29] Pastor-Fernandez I Pegg E Macdonald S Tomley FM Blake DP Marugan-Hernandez V. Laboratory growth and genetic manipulation of *Eimeria tenella*. Curr Prot Microbiol. (2019) 53:e81. 10.1002/cpmc.81 30811108

[30] Marugan-Hernandez V Cockle C Macdonald S Pegg E Crouch C Blake DP. Viral proteins expressed in the protozoan parasite *Eimeria tenella* are detected by the chicken immune system. Parasite Vector. (2016) 9:463. 10.1186/s13071-016-1756-2 27553200 PMC4994267

[31] Clark JD Billington K Bumstead JM Oakes RD Soon PE Sopp P. A toolbox facilitating stable transfection of *Eimeria* species. Mol Biochem Parasitol. (2008) 162:77–86. 10.1016/j.molbiopara.2008.07.006 18723051

[32] Thabet A Alnassan AA Daugschies A Bangoura B. Combination of cell culture and qPCR to assess the efficacy of different anticoccidials on *Eimeria tenella* sporozoites. Parasitol Res. (2015) 114:2155–63. 10.1007/s00436-015-4404-4 25773180

[33] PD Presidential Degree 56/2013 on Harmonization of the Directive 2010/63/EU, on the Protection of Animals Used for Scientific Purposes. Athens: Greek Government (2013).

[34] NRC Guide for the Care and use of Laboratory Animals. Washington, DC: National Academy Press (1996).

[35] Card RM Cawthraw SA Nunez-Garcia J Ellis RJ Kay G Pallen MJ. An *in vitro* chicken gut model demonstrates transfer of a multidrug resistance plasmid from Salmonella to commensal *Escherichia coli*. MBio. (2017) 8:e00777–17. 10.1128/mBio.00777-17 28720731 PMC5516254

[36] Bolder NM Microbial challenges of poultry meat production. Worlds Poult Sci J. (2007) 63:401–11. 10.1017/S0043933907001535

[37] Melkamu S Chanie M Asrat M Studies on coccidia in experimental infection with *Eimeria* spp in Rose-Cobb broiler chicken. J Anim Sci. (2017) 7:115–22. 10.5958/2277-940X.2017.00016.X

[38] Amer MM Awaad MHH El-Khateeb RM Abu-Elezz NMT Sherein-Said A Gheta MM Isolation and identification of *Eimeria* from field coccidiosis in chickens. J Am Sci. (2010) 6:1107–14. 10.7537/marsjas061010.128

[39] SPSS SPSS Statistics for Windows, Release 20.0. Armonk, NY: IBM (2018).

[40] Jin JZ Phytogenic feed additives: nutritional functions and mechanism of action in monogastric animals. Chin J Anim Nutr. (2010) 22:1154–64.

[41] Franz CM Baser KHC Hahn-Ramssl I Herbs and aromatic plants as feed additives: aspects of composition, safety, registration rules. In Florou-Paneri P Christaki E Giannenas I, editors. Feed Additives: Aromatic Plants and Herbs in Animal Nutrition and Health. London, UK: Academic Press (2019). p. 35–56. 10.1016/B978-0-12-814700-9.00003-0

[42] Giannenas I Florou-Paneri P Papazahariadou M Christaki E Botsoglou NA Spais AB Dietary oregano essential oil supplementation on performance of broilers challenged with *Eimeria tenella*. Arch Anim Nutr. (2003) 57:99–106. 10.1080/0003942031000107299 12866780

[43] Hockenhull J Turner A Reyher K Barrett D Jones L Hinchliffe S. Antimicrobial use in food-producing animals: a rapid evidence assessment of stakeholder practices and beliefs. Vet Rec. (2017) 181:510. 10.1136/vr.104304 28847873

[44] Hao H Cheng G Iqbal Z Ai X Hussain HI Huang L. Benefits and risks of antimicrobial use in food-producing animals. Front Microbiol. (2014) 5:288. 10.3389/fmicb.2014.00288 24971079 PMC4054498

[45] WHO Antimicrobial Resistance Global Report on Surveillance 2014. Geneva: World Health Organization (2014).

[46] Khalafalla RE Muller U Shahiduzzaman M Dyachenko V Desouky AY Alber G. Effects of curcumin (diferuloylmethane) on *Eimeria tenella* sporozoites *in vitro*. Parasitol Res. (2011) 108:879–86. 10.1007/s00436-010-2129-y 21057813

[47] Jitviriyanon S Phanthong P Lomarat P Bunyapraphatsara N Porntrakulpipat S Paraksa N. *In vitro* study of anticoccidial activity of essential oils from indigenous plants against *Eimeria tenella*. Vet Parasitol. (2016) 228:96–102. 10.1016/j.vetpar.2016.08.020 27692340

[48] Sikkema J de Bont JAM Poolman B. Mechanisms of membrane toxicity of hydrocarbons. Microbiol Rev. (1995) 59:201–22. 10.1128/MMBR.59.2.201-222.1995 7603409 PMC239360

[49] Weber FJ de Bont JAM. Adaptation mechanisms of microorganisms to the toxic effects of organic solvents on membranes. Biochim Biophys Acta. (1996) 1286:225–45. 10.1016/S0304-4157(96)00010-X 8982284

[50] Hessenberger S Schatzmayer G Teichmann K. *In vitro* inhibition of *Eimeria tenella* sporozoite invasion into host cells by probiotics. Vet Parasitol. (2016) 229:93–8. 10.1016/j.vetpar.2016.10.001 27809987

[51] WHO. National Policy on Traditional Medicine and Regulation of Herbal Medicines. Report of a World Health Organization Global Survey. Geneva: World Health Organization (2005).

[52] Ekor M. The growing use of herbal medicines: issues relating to adverse reactions and challenges in monitoring safety. Front Pharmacol. (2013) 4:177. 10.3389/fphar.2013.00177 24454289 PMC3887317

[53] Skoufos I Bonos E Anastasiou I Tsinas A Tzora A Effects of phytobiotics in healthy or disease challenged animals. In Florou-Paneri P. Christaki E Giannenas I, editors. Feed Additives: Aromatic Plants and Herbs in Animal Nutrition and Health. London, UK: Academic Press (2019). p. 311–37. 10.1016/B978-0-12-814700-9.00018-2

[54] Weber GM Michalczuk M Huyghebaert G Juin H Kwakernaak C Gracia MI. Effects of a blend of essential oil compounds and benzoic acid on performance of broiler chickens as revealed by a meta-analysis of 4 growth trials in various locations. Poult Sci. (2012) 91:2820–8. 10.3382/ps.2012-02243 23091138

[55] McDonald SE Nolan MJ Harman K Boulton K Hume DA Tomley FM Effects of *Eimeria tenella* infection on chicken caecal microbiome diversity, exploring variation associated with severity of pathology. PLoS ONE. (2017) 12:e0184890 10.1371/journal.pone.0184890 28934262 PMC5608234

[56] Voidarou C Bezirtzoglou E Alexopoulos A Plessas S Stefanis C Papadopoulos I. Occurrence of *Clostridium perfringens* from different cultivated soils. Anaerobe. (2011) 17:320–4. 10.1016/j.anaerobe.2011.05.004 21621626

[57] Tsiotsias A Voidarou C Skoufos I Simopoulos C Konstadi M Kostakis D Stress-induced alterations in intestinal microflora. Microb Ecol Health Dis. (2004) 16:28–31. 10.1080/08910600410028632

[58] Stanley D Geier MS Chen H Hughes RJ Moore RJ. Comparison of fecal and cecal microbiotas reveals qualitative similarities but quantitative differences. BMC Microbiol. (2015) 15:51. 10.1186/s12866-015-0388-6 25887695 PMC4403768

[59] Walter J. Ecological role of lactobacilli in the gastrointestinal tract: implications for fundamental and biomedical research. Appl Environ Microb. (2008) 74:4985–96. 10.1128/AEM.00753-08 18539818 PMC2519286

[60] Meehan CJ Beiko RG. A phylogenomic view of ecological specialization in the Lachnospiraceae, a family of digestive tract-associated bacteria. Genome Biol Evol. (2014) 6:703–13. 10.1093/gbe/evu050 24625961 PMC3971600

[61] Giannenas I Papaneophytou C Tsalie E Mavridis S Triantafillou E Kontopidis G. Effect of benzoic acid and essential oil compounds on performance of Turkeys, intestinal microbiota, intestinal morphology and antioxidant status. Asian Austral J Anim Sci. (2014) 27:225–36. 10.5713/ajas.2013.13376 25049947 PMC4093202

